# Helicobacter pylori infection manifesting as Hypereosinophilic syndrome and immune thrombocytopenia complicated by portal vein thrombosis and ischemic colitis

**DOI:** 10.1016/j.idcr.2022.e01451

**Published:** 2022-02-12

**Authors:** Awni Alshurafa, Mustafa Sied, Maab Elkhdier, Ahmed M. Abdalhadi, Mohamed A. Yassin

**Affiliations:** Hamad Medical Corporation, Doha, Qatar

**Keywords:** Hypereosinophilic syndrome, Immune thrombocytopenia, *H. Pylori*, Portal vein thrombosis

## Abstract

Hypereosinophilic syndromes (HES) are a group of uncommon disorders characterized by persistent eosinophils overproduction which can lead to tissue damage and organs dysfunction secondary to eosinophils tissue infiltration and inflammatory mediators’ release. Causes of secondary HES include parasitic infection, some solid tumors, underlying connective tissue disease, allergic conditions and T cell lymphoma. *Helicobacter pylori (H. pylori)* has been reported only once as a cause of secondary HES in the literature. We report the second case of *H. pylori* infection in 29-year-old male patient who presents with HES and secondary Immune thrombocytopenic purpura (ITP). This case is different from the first reported case by the presence of HES complication on presentation manifesting as portal vein thrombosis, which was further complicated by ischemic colitis. *H, pylori* eradication therapy alone was successful in a resolution of hypereosinophilia and platelets recovery without the need of corticosteroids or any other treatment.

## Introduction

Hypereosinophilia (HE) is defined as an absolute eosinophil count (AEC)> 1.5 × 109/L on two tests at least four weeks apart and/or evidence of tissue hypereosinophilia confirmed by pathologic examination. Hypereosinophilic syndrome (HES) is hypereosinophilia and organ dysfunction, which is related to tissue HE, given that other causes of organ damage has been excluded [1]. HES can be categorized as primary (clonal), secondary (reactive), or idiopathic. Causes of secondary HES include parasitic infection, some solid tumors, underlying connective tissue disease, allergic conditions, and T cell lymphoma [2], [3]. *Helicobacter pylori (H. pylori)* has been reported only once in the literature as a cause of secondary HES [4]. Immune thrombocytopenia (ITP) is an acquired immune medicated process characterized by transient or persistent thrombocytopenia [5]. ITP is a common bleeding disorder with reported incidence of 3.9 per 100,000 [6]. ITP is subclassified into primary and secondary. Secondary causes include autoimmune diseases, immunodeficiency syndromes, lymphoid malignancies, and underling infection. In contrast to HES, *H. pylori* is well reported as a secondary cause of ITP and it’s eradication results in platelets counts recovery [7], [8], [9]. Thromboembolism estimated to affect around 25% of HES patients, causing 5–10% mortality, and it can be the presenting complaint [10], [11], [12]. We report a case of a 29-year-old male patient who presented with abdominal pain and bloody diarrhea and was found to have *H. pylori*-related HES and secondary ITP complicated by portal vein thrombosis and ischemic colitis. *H. pylori* eradication alone results in platelets count recovery and eosinophils normalization without the need for any further treatment.

## Case report

A 29-year-old Bangladeshi male patient, previously healthy, presented to the Emergency Department complaining of generalized abdominal pain and bloody diarrhea for 10 days. He denied nausea, vomiting, fever, skin rash, anorexia, or weight loss. He is not known to have any chronic gastrointestinal disease or previous parasitic infection. Also, He denied any new drug intake or any allergic reaction. Family history is not significant for malignancy.

On General examination, the patient was vitally stable, with no documented fever, skin rash, or palpable lymphadenopathy. The abdominal examination showed generalized tenderness, no organomegaly, and normal bowel sounds. Other systems examination was unremarkable.

Complete blood counts showed Hemoglobin of 14.7 gm/dL (13.5–17.5 gm/dL), leukocytes of 23,500/L (4000–10,000/L) with absolute eosinophil count of 14,700/L (0–500/L), and platelets counts of 42,000/L (150,000–400,000/L).

Renal, liver function tests, lactate, and coagulation profile were in normal limits. Stool tested twice negative for ova and parasites. Peripheral smear reported twice as marked eosinophilia, mild lymphocytosis, and moderate thrombocytopenia, no blasts or atypical cells were seen. Chest X ray and echocardiography were unremarkable.

CT abdomen revealed portal vein thrombosis with diffuse concentric thickening of colonic segments involving hepatic flexure, transverse colon, splenic flexure up to the lower part of the descending colon reported as inflammatory versus ischemic colitis. The liver and spleen showed normal echotexture and size, and there was no lymphadenopathy.

The patient was admitted under the medical team for further evaluation and management. Based on his age, clinical presentation, and imaging, the initial impression was inflammatory bowel disease complicated by portal vein thrombosis, and he was started on IV hydration and anticoagulation.

**Fig. 1:**
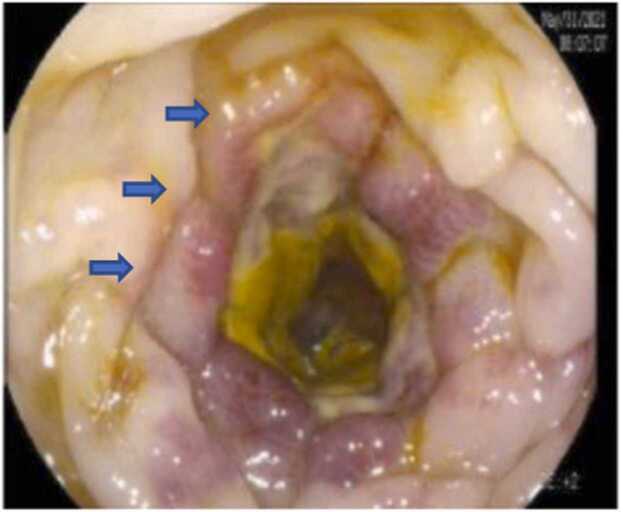
colonoscopy shows sharply defined edematous segmental erythema at proximal transverse colon to splenic flexure suggestive of ischemic colitis.

The gastroenterology team was consulted; they recommended colonoscopy.it was done and showed a picture of ischemic colitis reported as sharply defined edematous segmental erythema at the proximal transverse colon to the splenic flexure, and no gangrene was found (Fig. 1). A biopsy was taken from the transverse colon. The histopathological examination showed fragments of colonic mucosa with focal surface mucosa denudation, focal ulceration, and numerous eosinophils in the lamina propria.

In view of colonoscopy findings and sever eosinophilia, the hematology team was consulted to exclude underlying myeloproliferative disorder. Patient was tested negative for FIP1 L1–PDGFRa (FIP1–like 1 platelet‑derived growth factor receptor A), PDGFRB (Platelet Derived Growth Factor Receptor Beta), JAK2V617F, CALR and BCR-ABL mutations.

Also, he was investigated to rule out underlying thrombophilia. Factor V Leiden, prothrombin gene mutation, antinuclear antibody (ANA), anticardiolipin, lupus anticoagulant, and Beta-2 glycoprotein antibody tests came negative.

He was also evaluated for the other secondary causes of ITP. Hepatitis B, hepatitis C, HIV, and ANA results were negative. But *H. pylori* stool antigen came positive which was sent as part of thrombocytopenia work up.

The patient was started on *H. pylori* quadrable eradication regimen including Bismuth subcitrate, Tetracycline, Metronidazole, and proton pump inhibitor (PPI) for 14 days.

**Fig. 2:**
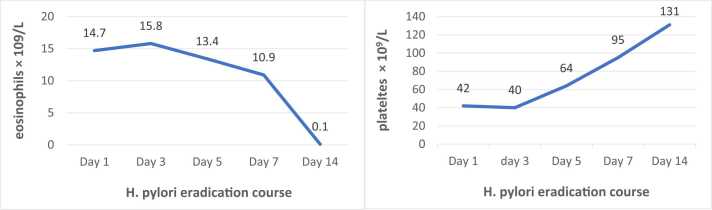
eosinophils and platelets response to *H. pylori* eradication therapy.

There was significant improvement in eosinophilia and thrombocytopenia after starting *H. pylori* treatment (see Fig. 2). Eosinophils dropped to 10.9 × 10^9^/L from 15.8 × 10^9^/L, and platelets increased to 95 × 10^9^/L from 40 × 10^9^/L after one week of treatment. Follow up CBC after two weeks showed eosinophils of 0.1 × 10^9^/L and platelets of 131 × 10^9^/L and patient was tolerating and complaint to anticoagulation. Given this dramatic improvement on *H. pylori* eradication, we concluded that the final diagnosis is *H. pylori‑*related HES, secondary ITP, portal vein thrombosis complicated by ischemic colitis was made.

The most challenging part in this patient’s management was the concomitant occurrence of portal vein thrombosis and ischemic colitis, which necessitate starting anticoagulation on one hand and thrombocytopenia which put him at high risk of bleeding on the other hand. Balancing the benefits and risks, we decided to keep the patient on prophylactic anticoagulation and when the platelets increased to more than 50,000/L, he was started on full anticoagulation.

## Discussion

Hypereosinophilic syndromes (HES) are a group of uncommon disorders characterized by persistent eosinophils overproduction which can lead to tissue damage and organs dysfunction secondary to eosinophils tissue infiltration and inflammatory mediators release [1]. Although HES can affect any organ in the body, the most commonly involved organs are the skin, lungs, heart, and gastrointestinal tract [13]. HES can be classified as primary (clonal), secondary (reactive), or idiopathic [2].

Clonal HES occurs in the setting of an underlying stem cell, eosinophilic, or myeloid neoplasm [2]. To diagnose clonal eosinophilia needs to demonstrate either a molecular/cytogenetic marker of clonality or bone marrow examination that is consistent with an otherwise classified myeloid malignancy [14]. Our patient had severe eosinophilia, thrombocytopenia, and thrombosis so we evaluated him for underlying primary HES by sending the FIP1L1–PDGFRA fusion gene, PDGFRB JAK2V617F mutation, CALR, and BCR‑ABL mutation. All tests came negative, and because of significant response to *H. pylori* eradication, we were able to rule out primary HES.

Infections cause hypereosinophilia by triggering Th2 cell-derived IL-5, IL-3, and GM-CSF. Eosinophils enhance immune responses by releasing cytokines and chemokines. While eosinophil mediated tissue inflammatory response by different mechanisms, it is involved in various microbial, such as viruses and parasites, antigens process and presentation[15], [16], [17].

*H. pylori* is a gram-negative bacterium that colonizes the gastric mucosa. it has been implicated in the development of peptic ulcer disease, gastric cancer, gastric mucosa-associated lymphoid tissue (MALT) lymphoma, unexplained iron deficiency anemia, and immune thrombocytopenia but it was only reported once as cause of secondary HES[4], [7]. Mangal et al. report the first case of *H. pylori* presenting with HES and secondary ITP in a 66-year-old male patient who presented with abdominal pain and symptomatic ITP, work up showed positive urea breath test and eosinophilic duodenitis. Anti‑*Helicobacter pylori* treatment alone results in clinical and hematological improvement.

The exact mechanism of *H. pylori*-induced ITP is still uncertain. However, there are different hypotheses. One of these hypotheses is molecular mimicry which means that our bodies produce antibodies in response to *H. pylori* infection leads to cross-reactivity with platelet surface antigens. Chronic *H. pylori* infection stimulates the immune system to produce non-specific autoantibodies. However, this does not explain autoimmune response in ITP patients. Also, it has been reported that *H. pylori*-infected patients have enhanced phagocytic capacity and low expression levels of inhibitory FcγRIIB in their circulating monocytes. Activation of dendritic cells and macrophages by *H. pylori* released components found to be responsible as well. Moreover, *H. pylori* can induce platelets aggregation by direct interaction of IgG antibodies and von Willebrand factor against with their compatible receptors, FcγRIIA and GPIb, on platelets [18].

In our patient, the presentation was different and more challenging as he presented with abdominal pain and bloody diarrhea and initial workup showed sever eosinophilia, thrombocytopenia, portal vein thrombosis and picture of ischemic Vs inflammatory colitis. The differential diagnosis at that point was infectious (especially parasitic), inflammatory colitis, thrombophilia or myeloproliferative disorder. *H. pylori* stool antigen was sent as part of ITP work up. However, when the patient showed improvement by a dramatic decrease in eosinophils and improvement in the platelets with *H. pylori* treatment, the final diagnosis was *H. pylori*-related HES and ITP.

Not only the diagnosis was challenging in this patient but also the management wasn’t straightforward. The presence of portal vein thrombosis and ischemic colitis indicate the use of anticoagulation to prevent further thrombosis progression and gangrene formation, but the high risk of bleeding secondary to thrombocytopenia was a concern.

Balancing the benefits and risks, we decided to keep the patient on prophylactic anticoagulation and when the platelets increased to more than 50,000/L, he was started on full anticoagulation.

## Conclusion,

we report the second case of *H. pylori* presenting with HES and secondary ITP. This case was different from the first reported case by the presence of HES complication on presentation manifesting as portal vein thrombosis which was further complicated by ischemic colitis*. H, pylori* eradication therapy alone was successful in resolution of hypereosinophilia and platelets recovery without the need of corticosteroids or any other treatment.

## Declaration

On behalf of all authors, the corresponding author states that there is no conflict of Interest.
